# A Program for At-Risk High School Students Informed by Evolutionary Science

**DOI:** 10.1371/journal.pone.0027826

**Published:** 2011-11-16

**Authors:** David Sloan Wilson, Richard A. Kauffman, Miriam S. Purdy

**Affiliations:** 1 Department of Biology, Binghamton University, Binghamton, New York, United States of America; 2 Department of Anthropology, Binghamton University, Binghamton, New York, United States of America; 3 Regents Academy, Binghamton City School District, Binghamton, New York, United States of America; American Museum of Natural History, United States of America

## Abstract

Improving the academic performance of at-risk high school students has proven difficult, often calling for an extended day, extended school year, and other expensive measures. Here we report the results of a program for at-risk 9th and 10th graders in Binghamton, New York, called the Regents Academy that takes place during the normal school day and year. The design of the program is informed by the evolutionary dynamics of cooperation and learning, in general and for our species as a unique product of biocultural evolution. Not only did the Regents Academy students outperform their comparison group in a randomized control design, but they performed on a par with the average high school student in Binghamton on state-mandated exams. All students can benefit from the social environment provided for at-risk students at the Regents Academy, which is within the reach of most public school districts.

## Introduction

Improving the performance of at-risk students is difficult at any age, but especially for at-risk teenagers, whose life challenges, personal habits, and social networks are often firmly entrenched [1], [2]. The problems posed by age are illustrated by the Promise Academy, a school associated with the Harlem Children's Zone, which started in 2004 with an incoming 1^st^ grade and 6^th^ grade class [3]. The students were selected by lottery, so they represented an at-risk community but not necessarily the most at-risk students within the community. Intensive efforts to improve academic performance, based on the same educational principles, succeeded for the 1^st^ graders but failed for the 6^th^ graders. The Promise Academy has since improved its success with the older students, but only with an intensive effort that includes an extended day, extended school year, meal and healthcare programs, and so on [4]. Other successful school programs for at-risk teenagers are similarly intensive (e.g., [5]–[7]).

Here we report a program for at-risk 9^th^ and 10^th^ graders in Binghamton, New York called the Regents Academy (RA). Students must have failed at least three of five courses during their previous year to qualify for the program, so they represent the most at-risk students within the community of Binghamton, rather than students selected by lottery from an at-risk community. The program is self-contained, with its own principal and teaching staff, and the cost per student is slightly greater than for the regular high school, but it takes place during the normal school day and year and similar programs are feasible for most public school districts. We present the results from the first year of the program, where RA students not only performed better than a comparison group that experienced the normal high school routine in a randomized control design, but they performed on a par with the average high school student.

### Using Evolutionary Science to Inform Educational Practice

Educational practices are informed by a variety of formal theories and informal rationales that are poorly integrated with each other [8]–[10]. The principles that inform the Regents Academy begin with the fact that schools are social groups whose members must cooperate with each other to achieve certain objectives. To design a successful school environment, it is important to understand how any social group achieves shared objectives, whatever they might be. In other words, educational practice must draw upon general theories of human social behavior.

The last few decades have witnessed a renaissance of theory and research on human social behavior [11], [12]. Many academic disciplines have taken part, but they converge upon three facts. First, the problem of how to cooperate to achieve shared goals is not restricted to the human species. It is encountered throughout nature. We must understand cooperation as a general evolutionary problem before we can properly understand it in our own species [13]. Economics has contributed to this synthesis as much as evolutionary biology, especially through the development of evolutionary game theory (e.g., [14], [15]).

Second, the human capacity for cooperation is based on our own evolutionary history, in addition to general evolutionary principles. We are uniquely cooperative among primates and unique among all species in our ability to cooperate among unrelated individuals. These distinctive abilities evolved by biocultural evolution, based on certain selective pressures, and resulted in a complex array of psychological mechanisms, including many that take place beneath conscious awareness. Our species-typical abilities to cooperate must be understood from both an ultimate (functional) and proximate (mechanistic) evolutionary perspective [16]–[18].

Third, knowledge derived from general evolutionary principles and our own evolutionary history can be used to enhance cooperation in real-world situations, such as a program for at-risk high school students. There is nothing static about cooperation. It succeeds under some environmental conditions and fails under others. We therefore evolved to be highly conditional in our willingness to cooperate with others, based on both conscious and unconscious psychological mechanisms. Since we are a cultural species that lives largely in a physical and mental world of our own making, we have tremendous latitude to construct social environments that favor cooperation as an evolutionarily successful strategy—but only if we make use of our knowledge [12].

One body of knowledge that we drew upon to design the Regents Academy is based on the work of Elinor Ostrom [19], [20], who received the Nobel Prize for economics in 2009. Ostrom is a political scientist by training but has become part of the evolutionary science community. Working primarily with groups attempting to manage common pool resources, she identified eight design features that contributed to the success of each group, which can also be used by groups attempting to achieve other shared objectives. Briefly, the design features are: 1) a strong group identity, including understanding and agreeing with the group's purpose; 2) benefits proportional to costs, so that the work does not fall unfairly on some individuals and unearned benefits on others; 3) consensus decision-making, since most people dislike being told what to do but will work hard to achieve their own goals; 4) low-cost monitoring, so that lapses of cooperation can be easily detected; 5) graduated sanctions to correct misbehaviors, which begin with friendly reminders and escalate only as needed; 6) conflict resolution that is fast and perceived as fair by group members; 7) sufficient autonomy for the group to make its own decisions without interference from other groups; 8) relations among groups that embody the same principles as the relations among individuals within the group. These design features are consilient with the general evolutionary dynamics of cooperation and the social environment of small-scale human societies throughout our own history as a species. Any educational program, including one for at-risk high school students, can potentially benefit from implementing these design features.

A second body of knowledge that we drew upon concerns development and psychological functioning, e.g., in benign vs. harsh environments [21]–[23]. The dysfunctions that arise from harsh environments are often interpreted as breakdowns of normal development and psychological functioning. While this is sometimes the case, evolutionary science offers an alternative possibility. Humans, like all species, are adapted to cope with harsh environments, but these adaptations involve tradeoffs with respect to long-term individual welfare and conduct toward others. Learning and cooperation to achieve long-term goals are eclipsed by the need to survive and reproduce over the short term. Some adaptations to harsh environments operate early in life and are difficult to reverse, such as the insecure attachment styles first documented by pioneering evolutionary psychologist John Bowlby [24], which has led to an extensive body of recent research [25]. Other mechanisms operate in response to immediate circumstances and can be modified by providing a safer and more secure environment [26], [27]. Most at-risk adolescents have experienced hardship throughout their lives, making it difficult for them to adapt to a safe and secure environment. Moreover, even if such an environment can be provided at school, the rest of their lives often remain harsh. Providing a safe and secure school environment might therefore not be *sufficient*, but it is surely *necessary* for at-risk students to cooperate and to achieve long-term goals.

A third body of knowledge that we drew upon concerns basic principles of learning that apply to many species [28], along with more specific adaptations for learning and cultural transmission in human groups [16], [29]–[34]. In a longitudinal study of students who were identified as gifted at the beginning of high school, Csikszentmihalyi et al. [35] examined the factors that led some to fulfill their promise and others to become merely average by the end of high school. It was primarily those who enjoyed what they were doing over the short term that developed their talents. The prospect of a long-term benefit, such as a career in science, was not sufficient to sustain day-to-day activities that were unrewarding. If this is true for the most gifted students, then it applies with even greater force for the most at-risk students [36]. If cooperation and learning outcomes aren't rewarding over the short term (what B.F. Skinner called “selection by consequences” [37]), positive outcomes cannot be expected over the long term. In addition, human groups evolved adaptations for social learning and spreading information for thousands of years before the advent of any formal school program. In modern times, complex bodies of information are culturally transmitted in hunter gatherer and many traditional societies largely without formal instruction [38]. Knowing how this occurs can help teachers shape their curriculum, instruction, and assessment to better maximize their students' natural tendencies to learn, and to make learning and teaching more spontaneous and self-organizing in modern classroom environments [39], [40].

## Methods

### Implementing the Design Features

It is important to distinguish between the functional design of a program and the implementation of the design in a real-world situation. Every design feature, such as creating a group identity, can be implemented in a number of ways, and the implementation that works best often depends upon local circumstances. In Ostrom's work on common pool resource groups, she was unable to predict performance outcomes in an analysis based on specific implementations, but she was able to predict performance outcomes in an analysis based on the presence or absence of design features [19]. The reason is easy to understand: if a particular design feature can be implemented in many ways, then the correlation between any single implementation and a successful outcome will be weak. When they are recognized as implementations of the same design feature, the correlation between the implementation of the design feature and a successful outcome can be strong.

Table 1 lists some ways that we attempted to implement the design features outlined in the previous section (for cooperative groups, psychological functioning in design vs. harsh environments, and basic principles of learning). Briefly, making the RA a self-contained program with its own principal, teaching staff and physical location helped to give it a group identity and provided a large degree of autonomy. Every effort was made to create a safe and nurturing environment. Students were consulted to establish the norms and practices of the school as much as possible. The principal was a large presence in the lives of the students, as a caring individual who also quickly enforced the rules. Short-term incentives were provided for cooperating and learning. Basic skills were taught to make up for the deficit of previous years. The schedule was designed to balance work with play to keep the school day enjoyable and to learn in the context of play. The material taught included, but did not obsessively focus upon, the state-mandated Regent exams. Parents and guardians of the students were engaged as much as possible, especially to praise the students for their positive performance.

**Table 1:** Design Features of the Regents Academy.

Design Principles for Cooperative Groups (numbers in parentheses refer to Ostrom design principle)	Psychological Functioning in Benign vs. Harsh Environments	Basic Principles of Learning
Self-contained program, with its own principal, teachers, support staff, and physical location (1, 4, 5).	Provided a safe and secure learning environment [33], [54], [55].	Evidence-based kernels: indivisible units of behavioral-change; and behavioral vaccines [56], [57].
First three days of school filled with group identity-building activities, council meetings/ group assemblies (1) [58]–[60].	Respect; cooperation; empathy taught as primary learning goals; encouraged through modeling [61]–[63].	Team competitions as behavior management programs, i.e. age-appropriate versions of the Good Behavior Game; emphasis on self-monitoring and engagement with school activities [53], [62], [64]–[70].
Every staff member interacted with every student every day (1, 3, 4).	Reduced bullying, stereotyping, and other negative behaviors [71]–[73].	Token economy and time-rewards for inhibition, including individual and group-level rewards [67], [74]–[77].
Pictures of students and staff engaged in school activities posted throughout building (1).	All staff members viewed nurturing and caring as primary roles [44], [78].	Small class size, ∼10∶1 student/teacher ratio [79]–[82].
Abundant praise (targeted at students and staff), with mild punishment (progressing in severity as conflict escalated) (2, 5) [83]–[89].	Pleasant greetings, with or without positive physical touch [90]–[93].	Thinking and reasoning skills developed with puzzles and games [94]–[97].
Rapid feedback about what works (4, 7).	Low-emotion or “private” reprimands [88], [98]–[100].	Public posting of achievements [84], [101]–[105].
Students consulted to establish the rules and practices of the school as much as possible (3).	Social workers, psychologists, counselors, etc. brought in when needed.	Art activities provided, including: mosaics, t-shirt design, and a student-painted mural in the hall [106]–[108].
Staff meetings twice per week (3, 4).	Incorporated play into daily routine [109]–[114].	**Evidence-based Teaching Strategies** [115]
All members of the RA, staff and students, were encouraged to provide feedback and develop new programs/ideas for the academy (3).	Parents contacted regularly to discuss student behavior (especially when good!) and to address any parent- or school-concerns [116]–[120].	Emphasis on common themes and fundamental principles in, and between, class subjects [121]–[125].
Principal was a large presence in the lives of the students, as a caring individual who also quickly enforced the rules (6).	Social development programs, e.g. Girl's Circle, a Crime Victims Assistance Center Inc. program designed to target challenges that young women are facing today: self-esteem issues, male/female issues, bullying, good decision making [126]–[128].	Teaching for understanding through: inquiry-based learning, Socratic discussion, discrepant events, demonstrations, and laboratory learning; challenging pre-/misconceptions, while building from experience [129]–[137].
Developed group constitution for all staff and students to sign at beginning of year (3) [60], [138].	Emphasis on written, numerical and science literacy with non-stigmatizing remedial courses [121], [122], [139]–[141].	Emphasis on student engagement; through choral response, peer-to-peer tutoring, cooperative learning, and engaging student interests [142]–[146].
Flexible schedule with the ability to modify on the basis of experience (4, 7).	Communal eating: breakfast and lunch provided daily, and healthy snacks made available throughout day [75], [147].	Learning progressions; with integrated classroom- and school-based assessment strategies ([148]–[152].
Excellent working relationship with district superintendant and other administrators (7, 8).	Emphasis on developing self-assessment, basic reasoning, and social skills [153]–[156].	Incorporating real-world relevance and current events into lessons [157], [158].
Mindfulness-based discipline & behavioral modification practices (5, 6, 7) [159]–[161].	Individualized one-on-one instruction/care provided when needed [162].	Integration among teachers; with lessons that spanned across curricula and classroom-based assessment strategies [163].

### Participants

Of the 117 9^th^ and 10^th^ graders who qualified for the RA by failing three or more courses during the previous year, 56 were randomly chosen to enter the program and the others were tracked as they experienced the normal routine at Binghamton's single high school (BHS). For all participants, the school district provided year in school, sex, ethnicity, class grades, Regents exam scores, and attendance records. All students were in either 9^th^ (*Freshman*) or 10^th^ grade (*Sophomores*) so year in school was coded as a dichotomous variable (“1” = Sophomore); sex was coded as a dichotomous variable (“1” = Female); and as BHS does not differentiate between *Whites* and *Hispanics*, and both the RA and comparison group were composed entirely of *Blacks* and *Whites*, ethnicity was coded as a dichotomous variable (“1” = Black); school attended (*Regents Academy* or *Comparison Group*) was coded as a dichotomous variable (“1” = Regents Academy).

### Measures

The outcome variables included quarterly grades in each core subject (math, science, English, and global studies) and the grades for all state-mandated Regents exams taken by the students at the end of the year, which include Algebra, Living Environment, Comprehensive English, and Global Studies. The Algebra and Living Environment exams were taken by all 9^th^ grade students and by the 10^th^ graders who had failed the exams during the previous year. English and Global Studies were taken by 10^th^ graders only. Class grades and exam scores range from 0 to 100. Class grades are not strictly comparable because the RA students and comparison group experienced different curricula and the grading standards might have been different. Regents exam grades therefore provide a more rigorous basis of comparison and are provided for the Binghamton High school as a whole in addition to the RA students and the comparison group.

It was not possible to assess each component of the RA, especially during its first year when the best implementations of the design features were being worked out. We do have preliminary data on the effects of absenteeism and a “fun club,” designed to engage the interests of the RA students, on academic performance (quarter averages). Absence rates were calculated as the sum of classes missed during the third and fourth quarters; participation in *fun club* was coded as a dichotomous variable (“1” = Participated in Fun Club).

We use an alpha level of 0.05 for all statistical tests. All missing data were excluded listwise from the analysis; running the analysis with imputed data did not affect the results. Three RA students and four BHS students in the comparison group were excluded from the analysis because they left partway through the year to attend an alternative education program (e.g. home schooling or BOCES, a trade-school alternative program); final N = 110.

## Results

Two RA and ten BHS students in the comparison group dropped out of school during the year, a difference that was statistically significant (χ^2^_110_ =  5.06, *p* = .024). Drop-outs were excluded from further analysis.

For the students who completed the school year, grades in the different class subjects were not significantly different within either the RA or BHS comparison groups (2×4 ANOVA, *F*(3, 378) = 2.29, *p* = .078), so we report the combined grade point averages as our outcome variable. Quarterly scores were used to calculate cumulative grade point averages, with the fourth quarter cumulative grade (i.e., the final average) being the overall performance indicator for the year. Table 2 displays the results of a sequential regression, analyzing the effects of demographic predictors (year in school, sex, and ethnicity), then treatment group, and then absence rate on student performance. Grade level, sex, and ethnicity did not significantly predict cumulative averages in any of the models (see table for values). An analysis of variance showed there to be no interaction between school (RA or BHS comparison group) and grade level (*F*(1,81) = 1.15, *p* = .287), sex (*F*(1,81)<.01, *p* = .949), or ethnicity (*F*(1, 81) = 2.70, *p* = .104) on cumulative averages, therefore, the average scores of 9^th^ and 10^th^ graders, males and females, and black and white students were not significantly different in either the RA or BHS comparison group.

**Table 2:** Results of Using Demographic Variables, School, and Attendance to Predict Final Averages of 2010-2011 School year.

Model	Predictor	*B* (*S*.*E*.)	β (partial *r*)	*p*
1	*Sophomore* a	−.719 (4.031)	−.020 (−.020)	.859
	*Female* a	−3.232 (4.11)	−.090 (−.088)	.434
	*Black* a	−2.852 (4.09)	−.080 (−.078)	.488
	***R*** ^2^ = .019			
2	*Sophomore* a	.510 (2.50)	.014 (.023)	.839
	*Female* a	−1.504 (2.56)	−.042 (−.067)	.557
	*Black* a	−1.702 (2.54)	−.048 (−.076)	.504
	*RA* a	28.086 (2.48)	.784 (.788)	< .001
	***R*** ^2^ = .628*			
3	*Sophomore* a	2.752 (1.96)	.077 (.158)	.164
	*Female* a	3.057 (2.07)	.085 (.166)	.144
	*Black* a	−3.403 (1.98)	−.095 (−.193)	.089
	*RA* a	30.220 (1.95)	.844 (.871)	< .001
	*Attendance* b	−.125 (.02)	−.418 (−.639)	< .001
	***R*** ^2^ = .883*			

Note: All missing cases excluded listwise; N = 83. Tolerance values for all variables in all models were >.86.

a -Dichotomous variable with ‘1’ equal to the variable's name.

b -Number of core classes missed in quarters 3 and 4.

*p<.001.

Adding school to the model resulted in the significant prediction of cumulative averages (*R* ^2^ = .628 (*R* ^2^ change = 0.610), *F* (4, 78) = 32.934, *p*<.001), where the students in the RA had averages that were 28 points higher than their BHS counterparts (*B* = 28.09, *SE* = 2.48, *p*<.001). This pattern of results suggests that over 60% of the variability in student scores is predicted by whether they were in the RA or comparison group.

**Figure 1:**
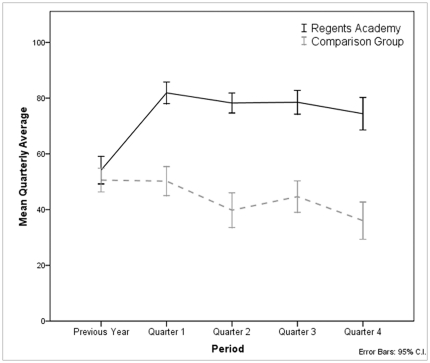
Grades for previous year and for each quarter of the academic year. Grades during the previous year were not significantly different between the RA (*M* = 54.97, *SD* = 17.31) and BHS comparison group (*M* = 52.58, *SD* = 15.47); t(95) =  −7.16, *p* = .476. Compared to the previous year, Regents Academy students increased their grades during the first quarter (*M* = 83.58, *SD* = 10.41; *t*(41) =  −12.791, *p* <.001) but not the comparison group (*M* = 52.83, *SD* = 17.01; *t*(47) =  −.095, *p* = .925). Grades decline slightly during the rest of the year. For both groups, the differences between the first and second quarter and the third and fourth quarter are statistically significant (paired sample t-test, *p*<.05).

Grades during the previous year were not significantly different between the RA (*M* = 54.97, *SD* = 17.31) and BHS comparison group (*M* = 52.58, *SD* = 15.47), as expected on the basis of the randomized design (Figure 1); *t* (95) =  −.72, *p* = .476. A large difference emerged by the first quarter and continued throughout the year, resulting in a mean final grade of 78.61 (*SD* = 12.61) for the RA students and 45.46 (*SD* = 16.06) for the BHS comparison group, *t*(95) = 11.345, *p*<.001. An analysis of GPA for each quarter shows that the highest grades were achieved during the first quarter and declined over the course of the year for both groups.

The regression analysis in Table 2 demonstrates that 9^th^ and 10^th^ graders, boys and girls, and whites and blacks benefitted equally from the RA. Although the Binghamton City School District records do not distinguish Hispanics as an ethnic category, within the RA, Hispanics (*M* = 84.78, *SD* = 6.35)) performed as well as other whites (*M* = 79.63, *SD* = 12.46) and blacks (*M* = 76.43, *SD* = 13.51); *F*(2,47) = 1.03, *p* = .366.

**Figure 2:**
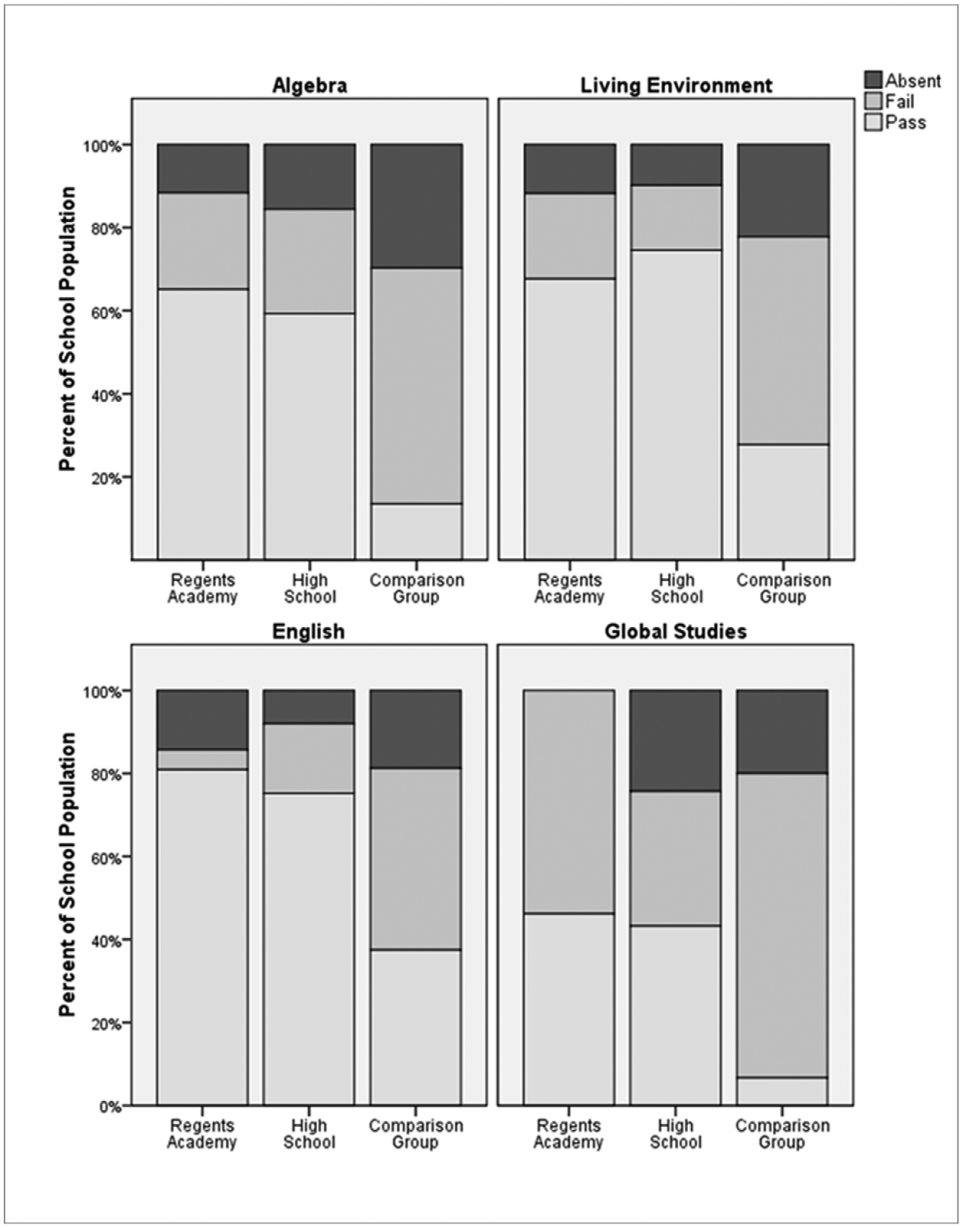
Performance on state-mandated exams in four subjects: Algebra, Living Environment, English, and Global Studies. Regents Academy (RA) students surpassed the passing rate of the comparison group on all subjects; Algebra: χ^2^ (1, N = 64) 18.33, *p*<.001; Living Environment: χ^ 2^ (1, N = 44) = 6.92, *p* = .017; English: χ^ 2^ (1, N = 31) = 9.19, *p* = .004; Global Studies: χ^ 2^ (1, N = 38) = 5.22, *p* = .030). There were no significant differences between the passing rates of RA students and the Binghamton High School (BHS) as a whole; Algebra: χ^ 2^ (1, N = 347) = .195, *p* = .711; Living Environment: χ^ 2^ (1, 370) = .673, *p* = .274; English: χ^ 2^ (1, 363) = 1.90, *p* = .218; Global Studies: χ^ 2^ (1, N = 434) = 1.19, *p* = .311. Students from the RA were more likely to attend the Global Studies exam than students from both the comparison group (χ^ 2^ (1, 41) = 5.61, *p* = .043) and BHS (χ^ 2^ (1, 565) = 8.226, *p* = .001). Attendance rates for the other exams did not differ.

The state-mandated Regents exams allow a rigorous comparison between the RA students, their comparison group, and Binghamton High School as a whole. In terms of pass rate, not only did the RA students outperform their comparison group, but they performed on a par with the average BHS student in all subjects (Figure 2: see figure caption for results of chi-square analyses). In terms of numerical grades, the RA students scored lower than the average BHS student in Living Environment (RA: *M* = 66.83, *SD* = 10.29; BHS: *M* = 77.34, *SD* = 13.09; *t*(368) =  −4.28, *p*<.001) and Global Studies (RA: *M* = 59.12, *SD* = 14.94; BHS: *M* = 66.85, *SD* = 16.25; *t*(432) =  −2.37, p = .018), but there was no significant difference in numerical grades for Algebra (RA: *M* = 67.79, *SD* = 10.86; BHS: *M* = 68.83, *SD* = 11.426; *t*(345) =  −.53, *p* = .596) and English (RA: *M* = 75.61, *SD* = 10.31; BHS: *M* = 74.79, *SD*  = 14.08; *t*(361) = .807, *p* = .807).

### Analysis of Design Components

Although the RA clearly succeeds as a whole program, it was not possible to assess each component, especially during the first year when the best implementations of the design features were being worked out. However, we do have preliminary data on the effects of absenteeism and a “fun club” designed to engage the interests of the RA students.

**Figure 3:**
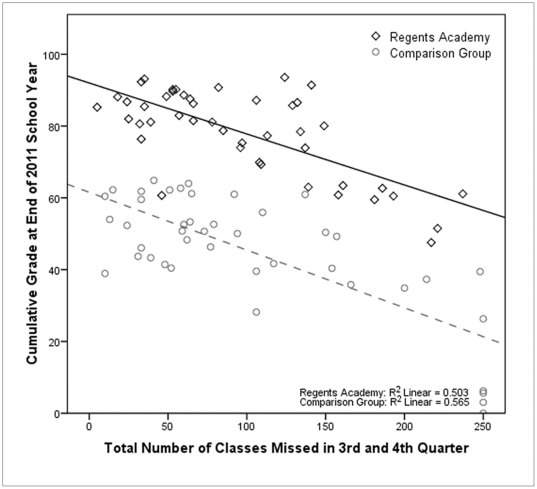
The effect of absenteeism on class grades for both RA students and BHS comparison group. Entering absence rates into the regression model identified attendance to be a predictor of academic performance (β = −.418, *t* = −7.291, *p*<.001; *R* ^2^ = .78, *R* ^2^ change  = .152, *F* = 54.60, *p* = .000). While there is a strong negative correlation between absence rates and grades for both schools (*r* = −.278, *n*  = 83, *p* = .011), this does not explain the difference between the two groups, since there is no correlation between attendance rate and experimental group (*r* = .119, *p* = .286), as RA students (*M*  = 96.91, *SD* = 60.36) did not miss more classes in the third and fourth quarters than the comparison group (*M* = 102.19, *SD* = 77.74); t (86) =  −.36, *p* = .724. Furthermore, controlling for the variability due to absences strengthened the correlation between school and academic success (*r* = .790, *p* <.001).

Figure 3 shows the effect of absenteeism on class grades for both RA students and the BHS comparison group. There is a strong negative correlation between absence rates and grades for both groups, but this does not explain the difference between the groups, since RA students did not have a lower absenteeism rate. When absence rate is entered into the regression model shown in table 1, it accounts for 15.2% of the variance and the total amount of variance explained by the model increases to 78% (*R* ^2^ = .780, *R* ^2^ change  = .152, *F* = 54.60, *p*<.001).

The fun club was initiated after the first quarter at the request of the students, who reported that they frequently couldn't relate to the class material and wanted to do things more closely aligned with their own interests. The teachers agreed to give up half of their class periods every Friday, providing a half-day for a “fun club”. The fact that they could make such a decision illustrates the importance of local autonomy (design principle #7). The students nominated a list of activities, illustrating our effort to include them in consensus decision-making as much as possible (design principle #3). We subsequently created a mosaic art class (thanks to a local artist who volunteered her services) and a “games group”, which provided an opportunity for students to learn how to juggle and play games that taught cognitive skills (offered by the 2nd author of this paper). The school was only able to provide a limited set of extracurricular programs during fun club, allowing some students to engage in activities aligned with their individual interests, while the others used the time to catch up on their work, go to the gym, or socialize.

**Figure 4:**
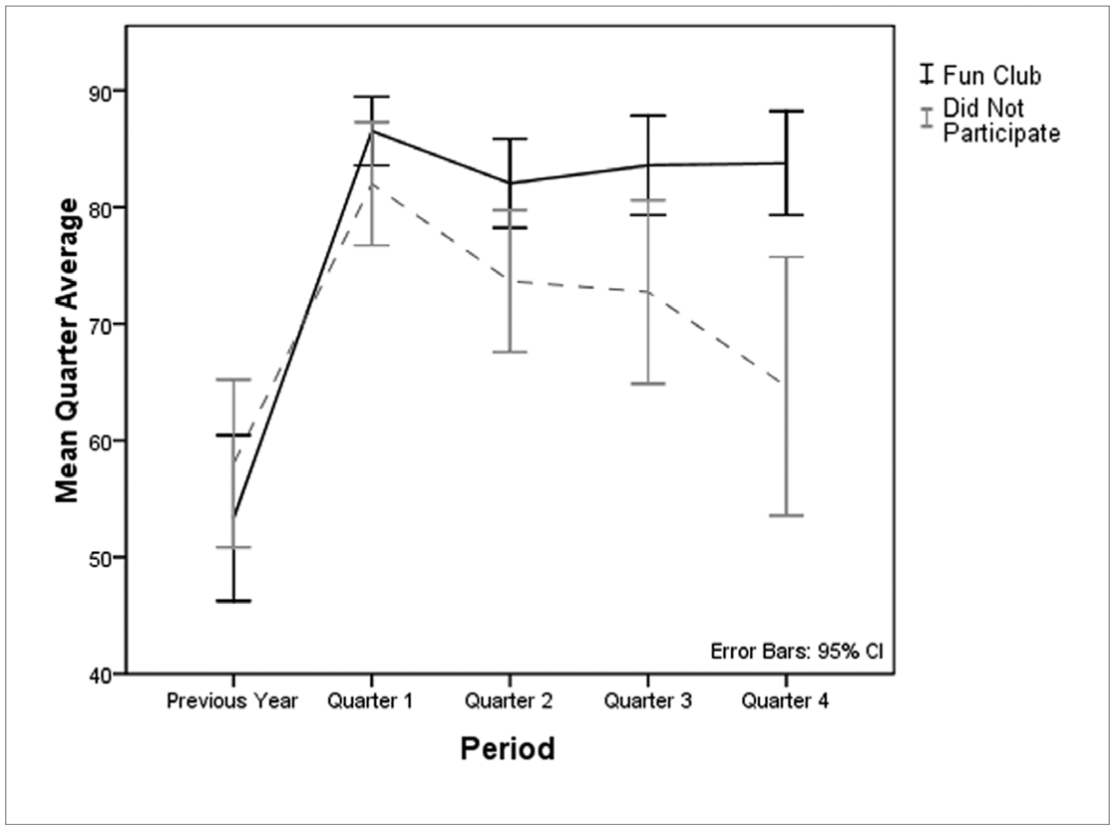
Grades of Regents Academy Students in Fun Club and of Those Who Did Not Participate. These two groups within the Regents Academy did not differ in their grades during the previous year (*t*(39) = −0.966, *p* = 0.340) or first quarter (*t*(40) = 1.519, *p* = 0.137), before fun club was implemented, but a widening gap appeared in the second quarter and continued through the rest of the school year; Qtr 2: *t(*40) = 2.37, *p* = .020; Qtr 3: t(40) = 2.46, *p* = .017; Qtr 4: *t*(40) = 3.21, *p* = .002.

When these two groups within the Regents Academy are compared (figure 4), they did not differ in their grades during the previous year (*t*(39) =  −0.966, *p* = 0.340) or first quarter (*t*(40) = 1.519, *p* = 0.137), before fun club was implemented, but a widening gap appeared in the second quarter and continued through the rest of the school year (see figure caption for details). The results of this quasi-experiment can be interpreted in a number of ways, but most of the interpretations point to the importance of making the school day fun and relevant to the interests of the students. The students who joined fun club had less time for studying their basic subjects, but earned higher grades for those subjects by virtue of their engagement with school.

## Discussion

Improving the academic performance of at-risk adolescents seems so difficult that it is sometimes regarded as a lost cause. One possibility is that academic performance has a large genetic basis [29], [41], [42]. Another possibility is that deficits that occur early in life are difficult or impossible to reverse [43]. Both of these cases imply that improvement is either impossible or requires heroic effort.

Evolutionary science presents a third possibility: The objective of school is to learn skills that will benefit the individual over the long term and society as a whole. This is a cooperative endeavor. Cooperation is an evolutionary strategy that succeeds under some circumstances and fails under others. Humans evolved to be facultative cooperators. Provide the wrong conditions, and cooperation becomes as difficult as if we had the wrong genes or have become permanently stunted. Provide the right conditions, and it becomes difficult to prevent people from cooperating.

Of course, environmental interventions for increasing the academic performance of at-risk adolescents have been proposed for decades, but they typically don't work [1], [7], which is why the problem appears so difficult. None of the design features that make up the RA are unusual, but evolutionary science provides a theoretical framework for bringing them together in a way that led to an unusual degree of success.

It is important to clarify how evolutionary science succeeded in identifying an effective collection of practices, where many other perspectives have failed. All educational practices have a surface logic; otherwise, no one would be tempted to implement them. If students aren't learning their basic subjects, it makes sense to concentrate more on the 3 R's and cut back on activities that seem superfluous, such as play, art, and sports. It seems efficient to create a school environment in which students interact primarily with others of the same age. It makes sense to implement no-touch rules to avoid the problem of sexual harassment. It makes sense to implement practices designed by experts without consulting the students. It makes sense to standardize practices and limit the opportunity of teachers to implement their own strategies. It makes sense to use monetary payment as an incentive to improve grades. It makes sense to quantify school and student performance in the form of test scores. All of these practices have a surface logic, but they don't always lead to positive outcomes. Worse, the unforeseen consequences of the practices are often diffuse and indirect, and therefore difficult to trace back to their source. Another problem with current educational policies is that they originate from many academic disciplines that are poorly integrated with each other. Even successful policies have difficulty spreading beyond their particular disciplinary boundaries.

Most experts who formulate educational policy accept evolution and assume that their ideas are consistent with current evolutionary science. Yet, only a few are actively drawing upon evolutionary science to formulate educational policy. One advantage of an explicitly evolutionary perspective is a single conceptual framework that transcends disciplinary boundaries. Once an educational program for at-risk youth is seen as a group that must cooperate to achieve a certain set of objectives, the general evolutionary dynamics of cooperation and the particular evolutionary history of our species become relevant. Insights from an academic discipline that is seldom associated with education (political science), involving groups that must cooperate to achieve very different objectives (managing common pool resources), become relevant to the general conceptual framework and available for a given application such as a school program for at-risk youth.

Using the general conceptual framework to formulate educational policy does not automatically result in a single set of practices guaranteed to work. Instead, it alters the perception of what appears reasonable or unreasonable. Some current practices continue to make sense but others, such as restricting play, causing children to interact primarily with others of the same age, no-touch rules, and rules imposed without regard to consensus decision-making, begin to appear problematic. New practices, or new combinations of old practices, become reasonable and even obvious in retrospect, although they were obscure from other perspectives. The new ideas that emerge from an explicitly evolutionary perspective are not guaranteed to work. Like all hypotheses, they must be tested in real-world applications. Science is always a dialectic between hypothesis formation and testing, and evolutionary science is no different.

Another insight from evolutionary science is that a well-functioning group is much like a well-functioning organism [12], [13]. Just as an organism has many organs and will die if any one of them is removed, a well-functioning group has numerous design features and will become dysfunctional if any one of them is removed. An educational program that includes most but not all of the design features is not good enough. This might explain the poor track record of environmental interventions that are not explicitly informed by evolutionary science.

The Regents Academy appears to have all the necessary “organs” to function well as a group. Each design feature makes intuitive sense, and it might seem that evolution isn't required to appreciate their utility—yet, they are frequently lacking in the average American high school social environment, especially from the perspective of at-risk students.

Even we didn't expect the RA to succeed so well that students who failed three or more classes during the previous year performed on a par with the average Binghamton high school student. This suggests an impressive degree of resilience in human development, such that adolescents who experienced hardship for most of their lives can still respond to an appropriately structured environment when it is provided, even for only one aspect of their lives (see also [4]).

Although the program as a whole was rigorously assessed in a randomized control design, future research will be required to assess its various components. According to the principal and teachers who work daily with the RA students, the most important ingredient is the provision of a safe, caring environment [44]. Once the students begin to regard school as a safe haven, they can switch from “survival” mode to “broaden and build” mode [27], [45], even when the rest of their lives remains harsh.

In addition to providing a safe haven, school must also provide short-term rewards for cooperating and learning skills that will be useful over the long term. Most species are extremely poor at learning tasks in which the costs are immediate and the benefits are deferred [46], [47]. Educational practice must reflect this basic fact about learning. Educational policies that make the school day less rewarding on a day-to-day basis in an effort to teach core skills are likely to backfire. Our results indicate that even half a day per week reserved for fun activities attuned to the students' interests can increase core academic performance.

Attendance had a strong effect on academic performance but does not explain the difference between the RA and comparison group. It is important to stress that attending school is not just a matter of wanting to for many at-risk high school students, who frequently skip school to earn money, care for siblings, and so on. Increasing attendance rate is an important future priority for both the RA and Binghamton High School as a whole.

A few other programs for at-risk high school students appear to have success rates approaching that of the Regents Academy, including the Sudbury Valley School [39], [40], Morningside Academy in Seattle [48],the Juniper Gardens Projects in Kansas City, Kansas [49], [50], a natural randomized-controlled study of London high schools by Rutter et al. ([51]), and a high school version of the Good Behavior Game [52], which was originally developed for elementary school classes [53]. A review of these programs reveals that they have largely converged on the practices that we have derived from an evolutionary perspective. These programs have not been widely copied, despite the fact that they work. Providing a general theoretical framework for *why* they work can help best practices spread faster.

The per student cost of the RA is slightly greater than for the average Binghamton high school student, but well within reach of the average public school district, especially when such positive outcomes can be expected. Indeed, the total societal benefit/cost ratio of a program that increases the academic performance of at-risk teenagers, measured in financial or any other terms, would be huge. In addition, the same design features that work for at-risk students can enhance the educational environment for all students. The limiting factor is not money but the lack of a clear sense of what to do. Evolutionary science can provide the entire field of education with a clearer sense of how to better manage the learning environment.
